# Hydrolyzed Milk-Derived Peptides Promote Erythropoietin Pathways and Hematologic Recovery: A Cross-Species Analysis

**DOI:** 10.3390/molecules30244739

**Published:** 2025-12-11

**Authors:** Liqing Zang, Akira Yokota, Misa Nakai, Kazutake Fukada, Norihiro Nishimura, Yasuhito Shimada

**Affiliations:** 1 Graduate School of Regional Innovation Studies, Mie University, Tsu 514-8507, Japan; nakai.innov@mie-u.ac.jp (M.N.); nishimura.norihiro@mie-u.ac.jp (N.N.); 2 Mie University Zebrafish Research Center, Tsu 514-8507, Japan; fukada@rohto.co.jp (K.F.); shimada.yasuhito@mie-u.ac.jp (Y.S.); 3 Department of Integrative Pharmacology, Mie University Graduate School of Medicine, Mie University, Tsu 514-8507, Japan; noissefnoc0526@gmail.com; 4 Rohto Pharmaceutical Co., Ltd., Osaka 544-8666, Japan

**Keywords:** bioactive peptides, cross-species model, EPO-iron axis, erythroid differentiation, hematopoietic regulation, nutraceutical

## Abstract

Anemia, characterized by reduced hemoglobin (Hb), remains a major health concern. Although iron and erythropoietin (EPO) therapies are effective, limitations in safety and accessibility have prompted interest in nutritional alternatives. Hydrolyzed milk-derived peptides (H-MDPs) contain bioactive sequences with diverse physiological effects, yet their role in erythropoiesis remains poorly defined. This study investigated the hematopoietic actions of H-MDP using zebrafish and mouse models. Adult zebrafish underwent phlebotomy-induced anemia and received oral H-MDP for 3 weeks. Hb levels, erythrocyte morphology, and expression of erythropoiesis- and iron-metabolism genes were assessed. In healthy mice, renal *Epo* expression, circulating EPO, and serum cytokines were measured after 2 weeks of H-MDP administration. H-MDP significantly accelerated Hb recovery in anemic zebrafish (4.6 ± 0.64 g/dL vs. 3.4 ± 0.66 g/dL in untreated fish at week 1) and markedly improved erythrocyte maturation. These effects coincided with strong induction of *epo*, *hif1aa/b*, *igf1*, *csf1a*, and *csf3b* in the heart and liver, as well as normalization of anemia-induced hepatic iron-transport genes (*tfa*, *fpn1*, *tfr2*) and reactivation of *hamp*. In mice, H-MDP elevated renal *Epo* mRNA and circulating EPO (approximately 2.3-fold) without altering steady-state Hb, and cytokine profiling with IPA-predicted activation of the erythropoietin signaling pathway. Collectively, these findings indicate that H-MDPs modulate erythropoiesis by coordinating the activation of EPO-related and iron-regulatory networks, supporting their potential as functional food ingredients for hematologic recovery and anemia management.

## 1. Introduction

Anemia, characterized by hemoglobin (Hb) levels below age- and sex-specific thresholds, remains a global public health challenge affecting both developing and developed countries [1]. It manifests as fatigue, dizziness, and impaired cognitive or physical performance, particularly in women of reproductive age and young children. In 2023, the World Health Organization estimated that approximately 605 million women aged 15–49 years had anemia, corresponding to a global prevalence of 30.7% [2]. Among women, iron deficiency anemia is especially prevalent owing to menstrual blood loss, which can gradually deplete iron stores and impair hemoglobin synthesis. Additional contributing factors, including inadequate dietary iron intake and increased demand during pregnancy, further exacerbate this condition. The etiology of anemia is multifactorial, encompassing deficiencies in iron, vitamin B_12_, and folate, as well as chronic inflammation, renal disease, blood loss, hemolytic disorders, and hemoglobinopathies [3,4]. Management strategies target the underlying cause and include iron or vitamin supplementation, treatment of bleeding or inflammation, and supportive interventions such as red blood cell transfusions or erythropoiesis-stimulating agents [3].

Erythropoiesis, the process by which red blood cells are generated from hematopoietic progenitors, is tightly regulated by genetic and hormonal mechanisms [5]. The key driver is erythropoietin (EPO), which binds to its receptor (EPOR) to promote survival, proliferation, and maturation of erythroid precursors. Iron is an essential cofactor for oxygen transport and cellular metabolism and is closely linked to erythropoietic activity [6]. In mammals, renal EPO stimulates red blood cell production, whereas hepatic hepcidin controls systemic iron availability by inhibiting the iron exporter ferroportin, thereby maintaining the balance between iron supply and erythropoietic demand [7,8]. In the body, most iron is recycled by macrophages, with dietary absorption compensating for only minor physiological losses. Ferritin is stored in hepatocytes and macrophages to buffer fluctuations, whereas regulatory factors such as erythroferrone fine-tune iron mobilization during stress erythropoiesis [9]. Thus, effective Hb restoration requires coordinated activation of both the EPO axis and iron-regulatory pathways. While recombinant EPO and iron therapies are clinically effective [10,11], their high cost, limited accessibility, and potential adverse effects have prompted growing interest in safer, well-tolerated oral bioactive compounds that can enhance erythropoiesis and iron utilization.

Bioactive milk-derived peptides (MDPs) are short amino acid sequences “encrypted” within casein and whey proteins and released during enzymatic hydrolysis, fermentation, or gastrointestinal digestion [12,13,14]. These peptides have garnered considerable attention as functional food components owing to their diverse biological activities, including anti-hypertensive [15], anti-obesity [16], antidiabetic [17], immunomodulatory [18], antioxidant [19], antimicrobial [17], and mineral-binding activities [18]. Hydrolyzed MDPs (H-MDPs) are proprietary hydrolysates composed of numerous short MDPs (typically 2–50 amino acids long) and are considered promising functional food ingredients or nutraceuticals. Importantly, H-MDPs differ from general MDPs in that they are produced through a controlled enzymatic process that yields a characteristic mixture of low-molecular-weight peptides (mainly <3 kDa). Several short peptide sequences (e.g., KHP, VRY, DIK, IKHQ) have been identified in the hydrolysate used in this study, although the full profile remains proprietary. This controlled composition distinguishes H-MDPs from naturally occurring or digestion-derived MDPs and may underlie distinct physiological effects. Despite the broad bioactivities reported for MDPs, their potential role in erythropoiesis remains largely unexplored, and it is not known whether they influence hematopoietic regulatory pathways, particularly in comparison with established nutritional interventions such as lactoferrin [19].

Zebrafish is a robust vertebrate model widely employed in developmental biology, drug discovery, and human disease studies [20,21]. It is particularly valuable for hematology and iron metabolism research, given the high conservation of erythropoietic pathways across vertebrates [22,23], including genes involved in hematopoietic specification, heme biosynthesis, and globin production [24]. Owing to this evolutionary conservation, zebrafish have emerged as a compelling model for investigating human anemia. Adult zebrafish exhibit both similarities and distinctive differences compared with mammals: definitive erythropoiesis occurs in the interstitium of the anterior kidney (head kidney) rather than in the bone marrow [25], while mature erythrocytes are nucleated and elliptical, facilitating direct morphological assessment [26]. EPO signaling is essential for both primitive and definitive erythropoiesis. In zebrafish, *epo* is primarily expressed in the heart and liver, with the kidney serving as the site of action. Anemia and hypoxia upregulate *epo* expression in the heart, highlighting its central role in systemic erythropoietic regulation [27].

In the current study, we established a phlebotomy-induced anemia model in adult zebrafish and evaluated its effect on hematologic recovery and erythrocyte morphology. Organ-specific expression of *epo*, its upstream regulators, and hepatic iron-related genes was analyzed to explore the underlying mechanisms. To determine translational relevance, healthy mice were orally administered H-MDP, and renal *Epo* mRNA, circulating EPO, serum ferritin, and cytokine profiles were measured. Collectively, these approaches were designed to elucidate the hematopoietic mechanisms of H-MDPs in vertebrate models.

## 2. Results

### 2.1. H-MDP Accelerates Hematologic Recovery in Phlebotomy-Induced Anemia in Zebrafish

**Figure 1:**
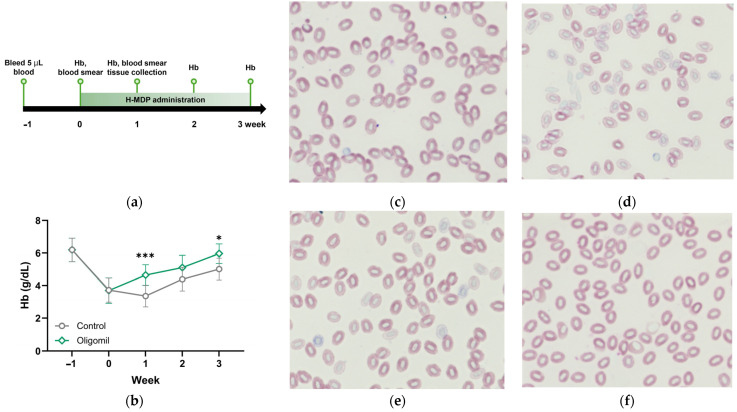
H-MDP facilitates hematologic recovery in a phlebotomy-induced anemia model in adult zebrafish. (**a**) Experimental timeline. Adult zebrafish were phlebotomized at week −1, and recovery was monitored for 4 weeks. From week 0, either an H-MDP-containing or a control diet was administered twice daily for 3 weeks. (**b**) Hb levels at each time point are shown in (**a**). Data are presented as mean ± standard deviation (SD) (*n* = 5–17). * *p* < 0.05, *** *p* < 0.0001 versus untreated anemic control. (**c**–**f**) Representative Giemsa-stained blood smears of (**c**) baseline normal control fish; (**d**) anemic control fish at week 0; (**e**) anemic control fish at week 1; and (**f**) anemic fish after 1 week of H-MDP treatment. H-MDP, hydrolyzed milk-derived peptides; Hb, hemoglobin.

We established a phlebotomy-induced anemia model in adult zebrafish to evaluate Hb levels and erythrocyte morphology during hematologic recovery. As illustrated in the experimental schema (Figure 1a), a single non-lethal blood draw (5 μL per fish) was performed, followed by a 1-week spontaneous recovery period. A second blood collection confirmed anemia at week 0, after which H-MDP was administered daily for 3 weeks. Hemoglobin levels were measured weekly throughout the experiment.

Phlebotomy markedly reduced Hb levels from 6.2 ± 0.32 g/dL (week −1) to 3.7 ± 0.26 g/dL at week 0 (Figure 1b). Prior reports have demonstrated that removal of ~5 μL of blood reliably induces anemia in adult zebrafish and results in hematologic depression lasting several days to weeks [28,29]. Consistent with these observations, our zebrafish also exhibited a substantial Hb drop, along with visible whole-body pallor and reduced swimming activity, phenotypic features characteristic of acute anemia. In untreated anemic zebrafish, Hb further declined to 3.4 ± 0.66 g/dL at week 1, whereas H-MDP treatment significantly increased Hb to 4.6 ± 0.64 g/dL (*p* < 0.0001). Hb levels remained higher in the H-MDP group throughout recovery (week 3: 6.0 ± 0.60 vs. 5.0 ± 0.68 g/dL, *p* < 0.05).

Cytological examination of Giemsa-stained erythrocytes further supported these findings. Healthy controls exhibited normochromic, densely stained erythrocytes (Figure 1c). At week 0, anemic fish displayed pronounced hypochromia with pale cytoplasmic staining (Figure 1d). Spontaneous recovery at week 1 partially restored chromasia in untreated fish (Figure 1e), whereas H-MDP–treated fish showed markedly improved erythrocyte coloration closely resembling healthy morphology (Figure 1f). These data indicate that H-MDP enhances both hemoglobin synthesis and erythroid maturation during recovery from anemia.

### 2.2. H-MDP Enhances Erythropoiesis- and Hypoxia-Related Gene Expression in Anemic Zebrafish

**Figure 2:**
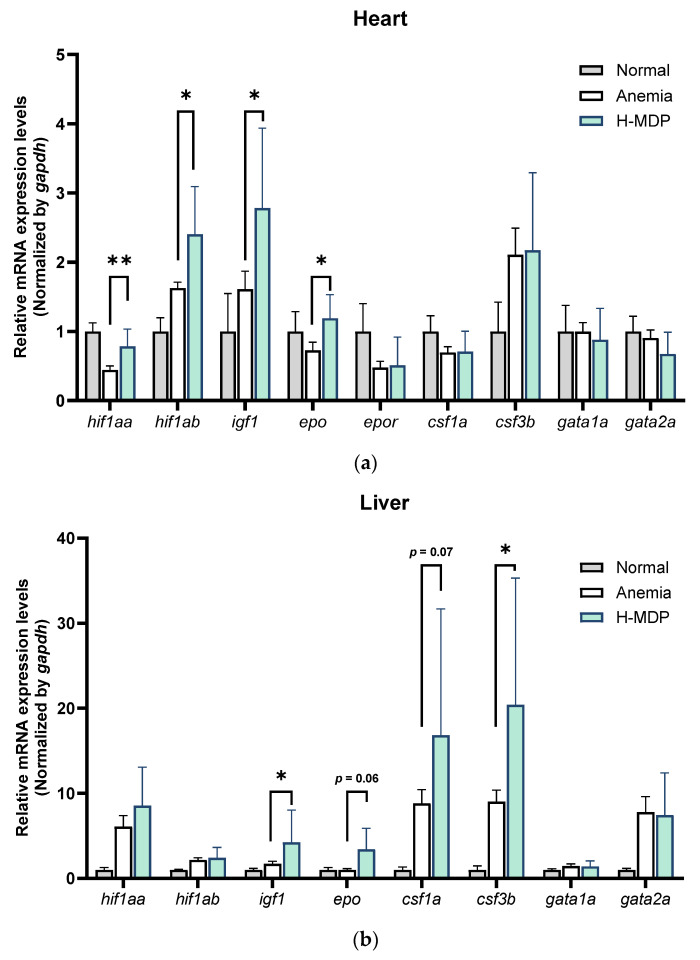

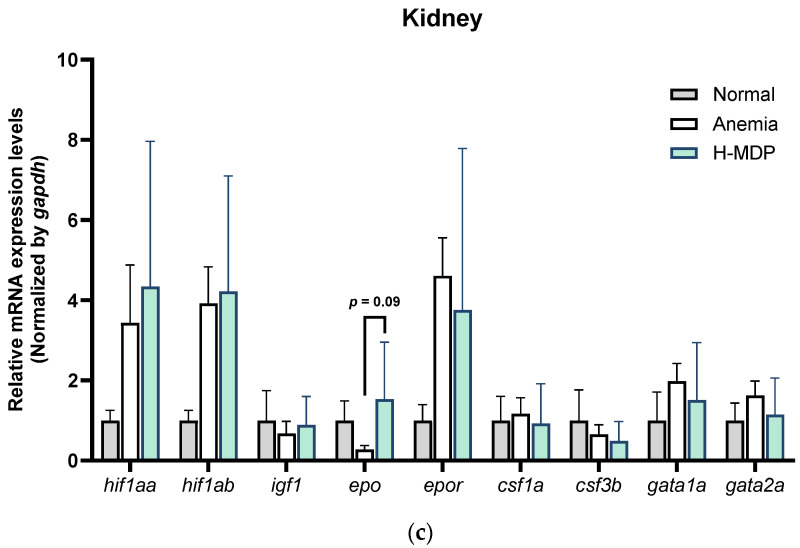
Expression levels of erythropoiesis- and oxygen-sensing genes in the heart (**a**), liver (**b**), and kidney (**c**) of adult zebrafish treated with or without H-MDP. Data are expressed as mean ± standard deviation (SD) (*n* = 6). * *p* < 0.05, ** *p* < 0.01 versus untreated anemic fish.

To elucidate the molecular mechanisms underlying the pro-hematopoietic activity of H-MDP, we analyzed the expression of erythropoiesis- and oxygen-sensing genes in the heart, liver, and kidney 1 week after phlebotomy. In the heart, an important *epo*-producing organ in zebrafish, H-MDP significantly upregulated the expression levels of *epo*, hypoxia-inducible factor 1 alpha a (*hif1aa*), hypoxia-inducible factor 1 alpha b (*hif1ab*), and insulin-like growth factor 1 (*igf1*) compared with the untreated anemic controls (*p* < 0.05, Figure 2a). These genes are closely associated with hypoxia signaling and erythroid progenitor proliferation, and their induction suggests that H-MDP enhances endocrine and paracrine drivers of erythropoiesis.

In the liver, another EPO-producing organ, H-MDP significantly increased the expression of *igf1* and colony-stimulating factor 3 (granulocyte) b (*csf3b*) (*p* < 0.05), while *epo* and colony-stimulating factor 1a (macrophage) (*csf1a*) showed upward trends relative to the anemic controls (*p* = 0.06 and *p* = 0.07, respectively) (Figure 2b). These genes support erythroid precursor expansion and macrophage-mediated iron recycling, suggesting a systemic pro-hematopoietic environment.

In contrast, the kidney—responsible primarily for EPO signaling rather than production in zebrafish—showed a modest, non-significant increase in *epo* (*p* = 0.09) and other genes (Figure 2c). Collectively, these findings suggest that H-MDP elicits a coordinated multi-organ transcriptional response, particularly in the heart and liver, to promote erythropoiesis and facilitate recovery from anemia.

### 2.3. H-MDP Modulates Hepatic Iron-Handling Gene Expression During Anemia Recovery

**Figure 3:**
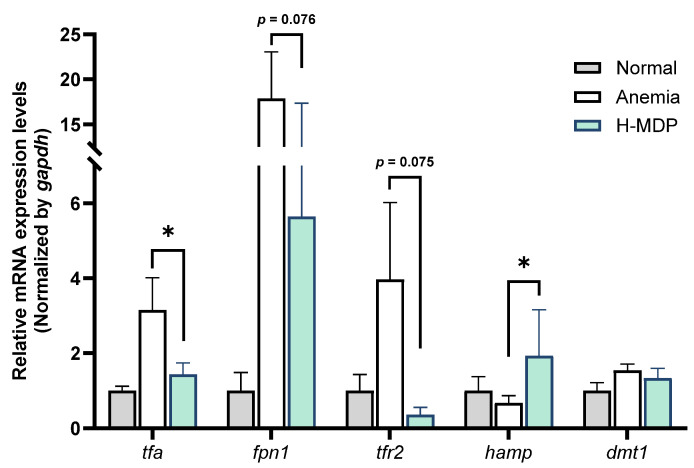
Expression levels of iron metabolism-related genes in the liver of adult zebrafish subjected to phlebotomy-induced anemia and/or H-MDP treatment for 1 week. Relative mRNA levels of *tfa*, *fpn1*, *tfr2*, *hamp*, and *dmt1* were quantified using qPCR and normalized to *gapdh*. Normal control: *n* = 6; Anemia: *n* = 8; H-MDP-treated: *n* = 8. Data are presented as mean ± standard deviation (SD). * *p* < 0.05 versus untreated anemic fish. H-MDP, hydrolyzed milk-derived peptides.

Because iron supply is essential for erythropoiesis, we evaluated hepatic expression of iron-related genes at week 1 post-phlebotomy. Anemia robustly increased expression of transferrin-a (*tfa*), solute carrier family 40 member 1 (*slc40a1* or *fpn1*), and transferrin receptor 2 (*tfr2*), reflecting activation of an iron-mobilization response to meet erythropoietic demand (*p* < 0.05, Figure 3). Concurrently, hepatic hepcidin (*hamp*) was markedly suppressed, consistent with the physiological requirement to facilitate iron release from hepatocytes and macrophages.

H-MDP administration significantly elevated *hamp* expression (*p* < 0.05) while reducing *tfa*, *fpn1*, and *tfr2* toward baseline expression levels (*tfa*, *p* < 0.05; *fpn1*, *p* = 0.076; *tfr2*, *p* = 0.075). This finding is consistent with partial normalization of the iron-regulatory program following anemia.

### 2.4. H-MDP Activates the Renal EPO Axis and Modulates Systemic Cytokine Signaling in Healthy Mice

**Figure 4:**
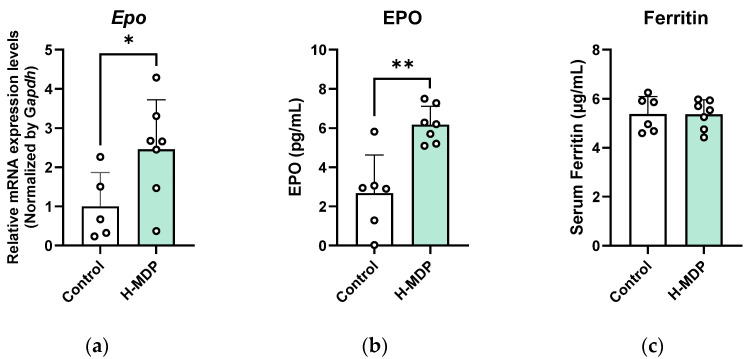
H-MDP stimulates the EPO axis in healthy mice. (**a**) Renal *Epo* mRNA levels after 2 weeks of H-MDP administration, quantified using qPCR and normalized to *Gapdh*. (**b**) Serum EPO concentration determined using ELISA. (**c**) Serum ferritin concentration determined using ELISA. Data are presented as mean ± standard deviation (SD). Control (*n* = 6) and H-MDP-treated (*n* = 7). *, *p* < 0.05, **, *p* < 0.01 vs. untreated controls. H-MDP, hydrolyzed milk-derived peptides; EPO, erythropoietin.

To evaluate cross-species relevance and determine whether H-MDP influences erythropoiesis under non-anemic conditions, healthy mice were administered H-MDP for two weeks. Renal *Epo* mRNA level was significantly increased in H-MDP–treated mice (*p* < 0.05), and circulating EPO levels were elevated by approximately 2.3-fold (*p* < 0.01; Figure 4a,b). In contrast, serum ferritin levels did not differ between the groups (Figure 4c), indicating that the systemic iron status was unaffected.

To further elucidate downstream signaling effects, serum cytokines were profiled using a mouse cytokine antibody array encompassing 96 analytes. While only five cytokines met a strict significance threshold of *p* < 0.05, a more permissive exploratory cutoff (|Log_2_ fold change| > 1.1 and *p* < 0.1) identified 13 differentially abundant proteins (Table 1). These candidates were subjected to Ingenuity Pathway Analysis (IPA), which integrates experimental datasets with curated molecular interaction networks to predict upstream regulators, canonical pathways, and functional outcomes [30,31].

**Figure 5:**
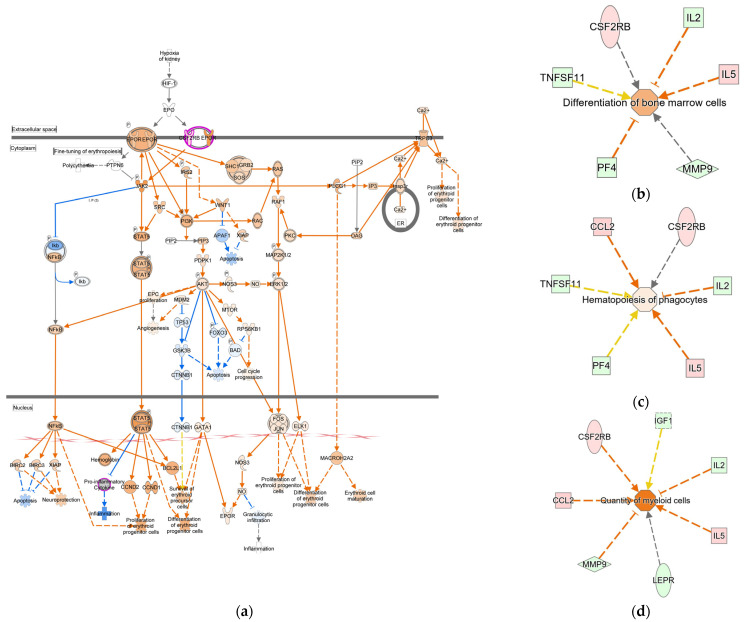
Serum cytokine pathways and function predictions after H-MDP administration in healthy mice. (**a**) The canonical erythropoietin signaling pathways predicted to be activated. (**b**–**d**). Diseases & Bio Function predictions indicate activation of “Differentiation of bone marrow cells” (**b**); “Hematopoiesis of phagocytes” (**c**); and “Quantity of myeloid cells” (**d**). Upregulated and downregulated cytokines are shown in red and green, respectively. Orange denotes predicted activation, blue denotes inhibition, and dashed lines represent indirect relationships. H-MDP, hydrolyzed milk-derived peptides.

IPA revealed activation of the EPO signaling pathway and inhibition of three other pathways: the tumor microenvironment pathway, the wound healing signaling pathway, and the estrogen receptor signaling pathway. Within the EPO pathway, IPA predicted the upregulation of EPOR and downstream activation of STAT and MAPK signaling cascades, which are key drivers of erythroid progenitor proliferation and differentiation [32] (Figure 5a). Furthermore, the IPA “Diseases & Bio Functions” module predicted activation of hematopoietic processes, including “Differentiation of bone marrow cells,” “Hematopoiesis of phagocytes,” and “Quantity of myeloid cells” (Figure 5b–d), aligning with the hematopoietic effects observed in zebrafish. Despite these signaling changes, no significant difference in Hb concentration was observed between the groups, indicating that H-MDP activated erythropoietic signaling pathways without disturbing steady-state Hb levels in healthy mice.

## 3. Discussion

In this study, H-MDP administration led to a markedly faster rebound of Hb levels and restoration of erythrocyte morphology compared with untreated anemic zebrafish (Figure 1). These physiological improvements were accompanied by coordinated transcriptional activation of erythropoietic pathways across multiple organs (Figure 2), suggesting that H-MDP enhances both erythroid maturation and the systemic response to hematologic stress. While the precise bioactive components responsible for this effect remain unknown, the findings collectively indicate that H-MDP acts as a nutritional modulator of erythropoiesis during recovery from blood-loss anemia.

In phlebotomized zebrafish, H-MDP induced a pro-erythropoietic transcriptional response across multiple organs (Figure 2). *Hif1α* is a key regulator of hypoxia-induced transcription that coordinates metabolic and iron-uptake programs to tissue hypoxia during anemia [33]. *Igf1*, a growth factor known to enhance erythroid proliferation via EPOR–JAK2–STAT5 and PI3K–AKT signaling, also stabilizes HIF through mTOR activation [34,35]. Additionally, *csf1a* (M-CSF) supports erythroblastic island macrophages and iron recycling, while *csf3b* (G-CSF) mobilizes marrow precursors and modulates the hematopoietic niche during stress [36]. In the heart, recognized as the principal site of EPO production in zebrafish, H-MDP administration significantly enhanced the expression of *epo*, *hif1aa*, *hif1ab*, and *igf1*, indicating the robust activation of erythropoiesis-related pathways. In the liver, which serves as a stress-inducible EPO source and a systemic endocrine organ, H-MDP upregulated the expression of *igf1*, *csf1a*, and *csf3b*, accompanied by a trend toward increased *epo* expression. Although the kidney is the primary site of definitive erythropoiesis in zebrafish, it exhibits relatively modest transcriptional changes, consistent with its role as an EPO target rather than a source. These synergistic, organ-specific responses likely contribute to enhanced erythroid maturation and survival during recovery from anemia, reflecting a “division of labor” in teleost hematopoiesis, distinct from mammals, where renal peritubular cells are the primary producers of EPO [23].

As the central regulator of systemic iron homeostasis, the liver exhibited a classical iron-mobilization response following phlebotomy. The expression of *tfa*, *fpn1*, and *tfr2* was markedly upregulated, whereas *hamp* expression was suppressed, reflecting the redistribution of iron for compensatory erythropoiesis (Figure 3). Administration of H-MDP reversed this pattern: expression of *hamp* was increased, whereas that of *tfa*, *fpn1*, and *tfr2* decreased toward baseline. Because hamp (hepcidin) elevation typically restricts iron export by suppressing ferroportin [37], this finding may appear counterintuitive during early anemia recovery. Thus, our interpretation that hamp induction represents a restoration of iron-regulatory negative feedback should be regarded as tentative. Without direct measurements of serum iron, transferrin saturation, ferroportin protein abundance, or hepatic iron content, the mechanistic implications of hamp upregulation remain unresolved. Nevertheless, the overall transcriptional pattern is consistent with partial normalization of hepatic iron handling and resembles the known effects of casein phosphopeptides (CPPs), which bind ferric iron, enhance iron solubility, and increase iron absorption [38,39]. These observations raise the possibility that H-MDP may influence iron mobilization through CPP-like activity. However, this remains hypothetical, as the present study did not characterize phosphorylated peptides within H-MDP or evaluate its iron-binding properties. Further biochemical validation will be required to clarify whether such mechanisms contribute to the observed hematopoietic effects.

In healthy mice, H-MDP elevated both renal *Epo* mRNA and circulating EPO protein levels, despite the absence of anemia, suggesting the stimulation of EPO production through HIF-independent mechanisms or IGF-regulated pathways (Figure 4). The cytokine array revealed selective, rather than broad, immunomodulation; Csf2rb and Ccl13 were upregulated, consistent with enhanced responsiveness to granulocyte-macrophage colony-stimulating factor and mild monocyte recruitment [40,41], whereas Igf1, Igf2, and Igfbp5 were downregulated, suggesting negative feedback within the IGF axis. These results indicate that H-MDP modulates hematopoietic and cytokine pathways in a species- and context-dependent manner rather than acting as a generalized inflammatory stimulant. Pathway-level analysis of differentially expressed cytokines revealed a hematopoietic signature (Figure 5). Canonical pathway enrichment predicted the activation of erythropoietin signaling, including the downstream JAK2–STAT5, MAPK, and PI3K–AKT cascades that promote erythroid progenitor proliferation and survival [42]. This aligns with the observed increase in renal *Epo* and serum EPO levels. Concurrent inhibition of the “Tumor Microenvironment”, “Wound Healing”, and “Estrogen Receptor” pathways suggests selective hematopoietic engagement while minimizing proinflammatory or proliferative responses. Functional predictions, including “Differentiation of bone marrow cells” and “Hematopoiesis of phagocytes”, further support early hematopoietic activation. The absence of an increase in Hb at this time point indicates that H-MDP primes erythropoietic signaling without inducing full erythropoiesis under steady-state conditions.

Our findings reveal a previously unrecognized hematopoietic function of H-MDP. While numerous studies have described broad bioactivities of milk-derived peptides—including antihypertensive, antioxidant, immunomodulatory, and mineral-binding effects—none have reported stimulation of erythropoiesis or coordinated activation of the EPO–HIF–IGF1 axis. Classical nutritional interventions such as lactoferrin [43,44,45] primarily act through iron sequestration, gut-immune regulation, or facilitation of intestinal iron absorption, but do not directly activate endocrine EPO production. In contrast, H-MDP elicited organ-specific transcriptional responses in zebrafish and increased renal Epo expression in mice, indicating a mechanistically distinct mode of action. Importantly, these effects may relate to the unique structural features of H-MDP, which is generated through controlled enzymatic hydrolysis that produces a reproducible mixture of low-molecular-weight peptides, including several sequences with potential bioactivity. This peptide composition distinguishes H-MDP from naturally occurring or digestion-derived MDPs and may underlie the erythropoietic and iron-regulatory responses observed. However, the functional contribution of individual peptides has not yet been defined, underscoring the need for future peptide profiling and targeted functional studies to elucidate the mechanisms underlying these hematopoietic effects.

## 4. Materials and Methods

### 4.1. Ethics Statement

All animal experiments were approved by the Ethics Committee of Mie University, performed in accordance with the Japanese Animal Welfare Regulation Act and Management of Animals (Ministry of the Environment of Japan), and complied with the international guidelines.

### 4.2. Preparation for H-MDP-Containing Diet for Zebrafish

H-MDP is a hydrolyzed milk-derived peptide (Oligomil^®^) produced by enzymatic digestion of casein-rich insoluble milk proteins using a mixture of neutral protease and neutral peptidase under controlled pH conditions (pH 5–8.5). After hydrolysis, the enzymes are heat-inactivated, followed by activated-carbon treatment, diatomaceous-earth filtration, vacuum concentration, sterilization, and spray-drying. Molecular weight profiling indicates that H-MDP consists predominantly of low-molecular-weight peptides (<3 kDa). Protein sequencing of the <2 kDa fraction confirmed the presence of multiple short peptides, including: KHP, VRY, DIK, KEK, IKHQ, KIHP, SryP, RYPS, KYIP, IHPF, MKPW, HQPHQ, VEQKH, KDERF, VDDKHY, DDKHYQ, KYKVPQ, VDDKHYQ, VDDKHYQK, and HKEMPFPKY. Due to proprietary restrictions, detailed enzymatic conditions and quantitative peptide composition cannot be disclosed.

To prepare a customized zebrafish diet, H-MDP was blended with wheat gluten powder (FUJIFILM Wako Pure Chemical, Osaka, Japan) at a concentration of 2.5% *w*/*w* using a previously described method [46]. Water was added to form a homogenous dough, which was then freeze-dried and ground into granules of an appropriate size for adult zebrafish consumption. The H-MDP-containing diet was stored at 4 °C and provided with freshwater during the experimental period.

### 4.3. H-MDP Administration to Phlebotomized Zebrafish

Wild-type AB strain zebrafish (*Danio rerio*) were obtained from the Zebrafish International Resource Center (Eugene, OR, USA) and bred in our laboratory according to the standard protocols outlined by ZFIN (https://zfin.atlassian.net/wiki/spaces/prot/overview; accessed on 13 August 2025). Phlebotomy was performed as previously reported [28,29]. Briefly, adult zebrafish (3-month-old) were anesthetized with 500 ppm 2-phenoxyethanol (FUJIFILM Wako Pure Chemical) and placed on a moist surface. Using a heparinized glass capillary connected to an aspirator tube, approximately 5 µL of blood was withdrawn via dorsal aorta puncture. The fish were then returned to freshwater for recovery. One week later, approximately 3 µL of blood was collected from the same individuals for Hb measurement. Zebrafish were then randomized into two groups: the anemia control group (fed a gluten-only diet) and the H-MDP group (fed the H-MDP-containing diet twice daily for 3 weeks at approximately 0.5 mg/g body weight/day). The Hb levels were assessed weekly.

### 4.4. Hb Measurement

The whole-blood Hb concentration was measured using the Hemoglobin B-Test Wako kit (FUJIFILM Wako Pure Chemical, PMDA document No. 16100AMZ04076000), following the manufacturer’s instructions. In brief, 2 μL of blood was mixed with 500 μL of a 10-fold diluted color-developing solution, incubated for 3 min at 25 °C, and the absorbance was measured at 546 nm and 660 nm using a spectrophotometer (Hitachi High-Tech, Tokyo, Japan). A standard curve was used to calculate the Hb concentration.

### 4.5. Giemsa-Stained Blood Smears

In brief, 5 µL of freshly collected blood was smeared onto glass slides and air-dried. The slides were fixed in methanol for 2 min and dried on a clean bench. A Giemsa working solution was prepared by mixing 1 mL of Giemsa stain (48900; Sigma-Aldrich, St. Louis, MO, USA) with 1 mL of M/15 phosphate buffer (pH 6.4). The slides were immersed in the staining solution for 60 min at room temperature in the dark, rinsed with distilled water for 15–30 s, air-dried, and mounted using Marinol (Muto Pure Chemicals, Tokyo, Japan). Erythrocyte morphology and staining characteristics were evaluated under a light microscope (BZ-X710; Keyence, Tokyo, Japan).

### 4.6. Real-Time Quantitative PCR (qPCR) Analysis

After 1 week of treatment, the liver, heart, and kidney tissues were harvested. Total RNA was extracted using the TRIzol reagent (Life Technologies, Carlsbad, CA, USA) and purified using an RNeasy Mini Kit (QIAGEN, Hilden, Germany). The RNA concentration and purity were assessed using a BioPhotometer (Eppendorf, Hamburg, Germany). Complementary DNA was synthesized from 300 ng of total RNA using a ReverTra Ace qPCR RT Kit (Toyobo, Osaka, Japan). qPCR was conducted using the Power SYBR Green Master Mix (Applied Biosystems, Foster City, CA, USA) on a StepOnePlus Real-Time PCR System (Applied Biosystems). For each target gene, the relative mRNA expression level was normalized to *gapdh*. Primer sequences are listed in Table 2.

### 4.7. Mouse Experiments

Seven-week-old male C57BL/6 mice (Japan SLC, Shizuoka, Japan) were acclimated for 1 week under standard housing conditions. Mice were randomly assigned to two groups: a control group (*n* = 6) and an H-MDP-treated group (*n* = 7). H-MDP was orally administered at a dose of 200 mg/kg body weight once daily for 14 consecutive days. Control mice received an equivalent volume of vehicle (distilled water). At the end of the treatment period, animals were euthanized, and whole blood was collected via cardiac puncture for subsequent analyses.

### 4.8. Mouse EPO and Ferritin Measurement

Whole blood samples were centrifuged at 4000× *g* for 15 min at 4 °C to isolate serum, which was then stored at −80 °C until analysis. Serum EPO levels were quantified using a Mouse Erythropoietin SimpleStep ELISA Kit (ab270893; Abcam, Cambridge, MA, USA). Serum ferritin levels were determined using a Mouse Ferritin (FTL) ELISA Kit (ab157713; Abcam), following the manufacturer’s instructions.

### 4.9. Mouse Cytokine Profiling and Pathway Analysis

Serum cytokine profiling was performed using the Mouse Cytokine Antibody Array C1000 (AAM-CYT-1000-8; RayBiotech, Norcross, GA, USA), according to the manufacturer’s protocol. Chemiluminescent signals were detected using an Amersham ImageQuant 800 system (GE Healthcare, Chalfont St. Giles, UK). Spot intensities were quantified using the ImageQuant TL software version 8.1 (GE Healthcare). Differentially expressed cytokines were analyzed using IPA (QIAGEN, Hilden, Germany) to identify relevant canonical pathways and upstream regulators.

### 4.10. Statistical Analysis

Data are presented as mean ± standard deviation. Statistical comparisons between two groups were performed using Student’s *t*-test. For multiple group comparisons, a one-way analysis of variance was performed, followed by Bonferroni’s post hoc test. A *p*-value < 0.05 was considered statistically significant. Data analyses were conducted using the GraphPad Prism version 10.6.0 (GraphPad Software Inc., San Diego, CA, USA).

## 5. Conclusions

This study demonstrated that H-MDP promotes hematologic recovery following blood loss and modulates erythropoietic and iron-regulatory pathways across species. In anemic zebrafish, H-MDP accelerated Hb restoration, promoted erythrocyte maturation, and upregulated genes associated with EPO signaling, hypoxia response, and iron metabolism. In healthy mice, H-MDP increased renal *Epo* expression and circulating EPO levels, accompanied by selective cytokine changes consistent with early hematopoietic activation. These findings indicate that H-MDP functions as a nutritional modulator by coordinating the regulation of EPO and iron homeostasis. Further characterization of its active peptide components and their mechanisms of action will clarify its potential as a functional food or nutraceutical ingredient in the management of anemia and hematologic health.

## Figures and Tables

**Table 1:** Differentially expressed cytokines identified in the serum of H-MDP-treated mice compared with vehicle control.

Symbol	Full Name	Log (Fold Change)	*p*-Value
CCL13	C–C motif chemokine ligand 13	0.17	0.001
IGF1	Insulin-like growth factor 1	−0.68	0.01
IGF2	Insulin-like growth factor 2	−1.19	0.02
IGFBP5	Insulin-like growth factor binding protein 5	−0.31	0.03
CSF2RB	Colony-stimulating factor 2 receptor subunit beta	0.37	0.03
IL5	Interleukin 5	0.44	0.06
LEPR	Leptin receptor	−0.17	0.08
CCL2	C–C motif chemokine ligand 2	0.48	0.08
PF4	Platelet factor 4	−0.25	0.09
CXCL16	C–X–C motif chemokine ligand 16	−0.17	0.09
MMP9	Matrix metallopeptidase 9	−1.89	0.09

**Table 2:** Primer sequences used in this study for qPCR.

Species	Gene Symbol	Forward Primer Sequence	Reverse Primer Sequence
Zebrafish	*hif1aa*	CTCGAGACCATACCGCTGTC	GCAACATTGGATGGCAGCAA
	*hif1ab*	CCACCACCCAAAAACTCCCT	GGAGTGGGGGCGATAAAACA
	*igf1*	AGTGTACCATGCGCTGTCTC	AATAAAAGCCCCTGTCTCCA
	*epo*	GGCCAGGCTTTCAATAAGG	TCACAGATGGGGCGTAATG
	*epor*	TCTCCGTGATGGTCAGATGCT	CTTCCCCGAGCTCCAGACT
	*csf1a*	ACTGTGCCCAGAGCAGCTTT	CCTCACAGTTCCAGTCCACAGA
	*csf3b*	GTGTGCAGCGGATGCTCAT	CTGCGAGGTCGTTCAGTAGGTT
	*gata1a*	CAGTTCAGCAGCGCTCTATTCA	AGCCTCAGGTGGCGAAAGT
	*gata2a*	CCACTGCAAGAATGGACGAA	GCCAAGCTTCCCCGAAGA
	*tfa*	TTACATGGGAGGGTCCTAATGAG	GGACACAACTGCTCGAGAAGAA
	*fpn1*	CTGGTAGCCCTTTCGATCTC	AATCGGGGGTTCAGTTGTAG
	*tfr2*	AAGAACATTCAGCAACAT	CAACATTCCCAAACTCTC
	*hamp*	CCTGGCTGCTGTCGTCAT	TGGTTCTCCTGCAGTTCTTCAC
	*dmt1*	GCAGCAATAAGAAGGAGGTGAA	CCACGAACACATTGATGAGGAAG
Mouse	*Epo*	CCACCCTGCTGCTTTTACTC	CTCAGTCTGGGACCTTCTGC
	*Gapdh*	GTTGTCTCCTGCGACTTCA	GGTGGTCCAGGGTTTCTTA

## Data Availability

All of the relevant data are presented within the paper.

## References

[1] WHO (2011). Haemoglobin Concentrations for the Diagnosis of Anaemia and Assessment of Severity; Vitamin and Mineral Nutrition Information System; WHO/NMH/NHD/MNM/11.1.

[2] WHO (2025). Global Anaemia Estimates: Key Findings.

[3] DeRossi S.S., Raghavendra S. (2003). Anemia. Oral Surg. Oral Med. Oral Pathol. Oral Radiol. Endodontol..

[4] Hess S.Y., Owais A., Jefferds M.E.D., Young M.F., Cahill A., Rogers L.M. (2023). Accelerating action to reduce anemia: Review of causes and risk factors and related data needs. Ann. New York Acad. Sci..

[5] Tsiftsoglou A.S., Vizirianakis I.S., Strouboulis J. (2009). Erythropoiesis: Model Systems, Molecular Regulators, and Developmental Programs. IUBMB Life.

[6] Mu Q.D., Chen L.Y., Gao X.T., Shen S.Y., Sheng W.J., Min J.X., Wang F.D. (2021). The role of iron homeostasis in remodeling immune function and regulating inflammatory disease. Sci. Bull..

[7] Ganz T. (2019). Erythropoietic regulators of iron metabolism. Free Radic. Biol. Med..

[8] Ganz T., Nemeth E. (2012). Iron metabolism: Interactions with normal and disordered erythropoiesis. Cold Spring Harb. Perspect. Med..

[9] Sposi N.M., Munshi A. (2015). Interaction between Erythropoiesis and Iron Metabolism in Human β-thalassemia—Recent Advances and New Therapeutic Approaches. Inherited Hemoglobin Disorders.

[10] Levy A.T., Weingarten S.J., Robinson K., Suner T., McLaren R.A., Saad A., Al-Kouatly H.B. (2025). Recombinant erythropoietin for the treatment of iron deficiency anemia in pregnancy: A systematic review. Int. J. Gynecol. Obstet..

[11] Macdougall I.C. (2024). Anaemia in CKD-treatment standard. Nephrol. Dial. Transpl..

[12] Korhonen H. (2009). Milk-derived bioactive peptides: From science to applications. J. Funct. Foods.

[13] Mohanty D.P., Mohapatra S., Misra S., Sahu P.S. (2016). Milk derived bioactive peptides and their impact on human health—A review. Saudi J. Biol. Sci..

[14] Kashung P., Karuthapandian D. (2025). Milk-derived bioactive peptides. Food Prod. Process. Nutr..

[15] Takano T. (2002). Anti-hypertensive activity of fermented dairy products containing biogenic peptides. Antonie Van Leeuwenhoek.

[16] Jakubowicz D., Froy O. (2013). Biochemical and metabolic mechanisms by which dietary whey protein may combat obesity and Type 2 diabetes. J. Nutr. Biochem..

[17] Corrêa J.A.F., Nazareth T.D., da Rocha G.F., Luciano F.B. (2023). Bioactive Antimicrobial Peptides from Food Proteins: Perspectives and Challenges for Controlling Foodborne Pathogens. Pathogens.

[18] Meisel H., FitzGerald R.J. (2003). Biofunctional peptides from milk proteins: Mineral binding and cytomodulatory effects. Curr. Pharm. Design.

[19] Hao L., Shan Q., Wei J., Ma F., Sun P. (2019). Lactoferrin: Major Physiological Functions and Applications. Curr. Protein Pept. Sci..

[20] Adhish M., Manjubala I. (2023). Effectiveness of zebrafish models in understanding human diseases-A review of models. Heliyon.

[21] Patton E.E., Zon L.I., Langenau D.M. (2021). Zebrafish disease models in drug discovery: From preclinical modelling to clinical trials. Nat. Rev. Drug Discov..

[22] Stachura D.L., Traver D., Detrich H.W., Westerfield M., Zon L.I. (2016). Chapter 2—Cellular dissection of zebrafish hematopoiesis. Methods in Cell Biology.

[23] Nikinmaa M. (2020). Environmental regulation of the function of circulating erythrocytes via changes in age distribution in teleost fish: Possible mechanisms and significance. Mar. Genom..

[24] Kulkeaw K., Sugiyama D. (2012). Zebrafish erythropoiesis and the utility of fish as models of anemia. Stem Cell Res. Ther..

[25] Amatruda J.F., Zon L.I. (1999). Dissecting hematopoiesis and disease using the zebrafish. Dev. Biol..

[26] Ransom D.G., Haffter P., Odenthal J., Brownlie A., Vogelsang E., Kelsh R.N., Brand M., vanEeden F.J.M., FurutaniSeiki M., Granato M. (1996). Characterization of zebrafish mutants with defects in embryonic hematopoiesis. Development.

[27] Paffett-Lugassy N., Hsia N., Fraenkel P.G., Paw B., Leshinsky I., Barut B., Bahary N., Caro J., Handin R., Zon L.I. (2007). Functional conservation of erythropoietin signaling in zebrafish. Blood.

[28] Zang L., Shimada Y., Nishimura Y., Tanaka T., Nishimura N. (2013). A novel, reliable method for repeated blood collection from aquarium fish. Zebrafish.

[29] Zang L., Shimada Y., Nishimura Y., Tanaka T., Nishimura N. (2015). Repeated Blood Collection for Blood Tests in Adult Zebrafish. J. Vis. Exp..

[30] Kramer A., Green J., Pollard J., Tugendreich S. (2014). Causal analysis approaches in Ingenuity Pathway Analysis. Bioinformatics.

[31] Zang L., Saitoh S., Katayama K., Zhou W., Nishimura N., Shimada Y. (2024). A zebrafish model of diabetic nephropathy shows hyperglycemia, proteinuria and activation of the PI3K/Akt pathway. Dis. Model. Mech..

[32] Bhoopalan S.V., Huang L.J., Weiss M.J. (2020). Erythropoietin regulation of red blood cell production: From bench to bedside and back. F1000Research.

[33] Kaplan J.M., Sharma N., Dikdan S. (2018). Hypoxia-Inducible Factor and Its Role in the Management of Anemia in Chronic Kidney Disease. Int. J. Mol. Sci..

[34] Muta K., Krantz S.B., Bondurant M.C., Wickrema A. (1994). Distinct roles of erythropoietin, insulin-like growth factor I, and stem cell factor in the development of erythroid progenitor cells. J. Clin. Investig..

[35] Hsieh H.H., Yao H., Ma Y., Zhang Y., Xiao X., Stephens H., Wajahat N., Chung S.S., Xu L., Xu J. (2022). Epo-IGF1R cross talk expands stress-specific progenitors in regenerative erythropoiesis and myeloproliferative neoplasm. Blood.

[36] Tichil I., Mitre I., Zdrenghea M.T., Bojan A.S., Tomuleasa C.I., Cenariu D. (2024). A Review of Key Regulators of Steady-State and Ineffective Erythropoiesis. J. Clin. Med..

[37] Nemeth E., Ganz T. (2021). Hepcidin-Ferroportin Interaction Controls Systemic Iron Homeostasis. Int. J. Mol. Sci..

[38] Walters M.E., Esfandi R., Tsopmo A. (2018). Potential of Food Hydrolyzed Proteins and Peptides to Chelate Iron or Calcium and Enhance their Absorption. Foods.

[39] Miquel E., Alegría A., Barberá R., Farré R. (2006). Casein phosphopeptides released by simulated gastrointestinal digestion of infant formulas and their potential role in mineral binding. Int. Dairy J..

[40] Tsiftsoglou A.S. (2021). Erythropoietin (EPO) as a Key Regulator of Erythropoiesis, Bone Remodeling and Endothelial Transdifferentiation of Multipotent Mesenchymal Stem Cells (MSCs): Implications in Regenerative Medicine. Cells.

[41] Li L.F., Dai F., Wang L.L., Sun Y.T., Mei L., Ran Y., Ye F.C. (2023). CCL13 and human diseases. Front. Immunol..

[42] Richmond T.D., Chohan M., Barber D.L. (2005). Turning cells red: Signal transduction mediated by erythropoietin. Trends Cell Biol..

[43] Ianiro G., Niro A., Rosa L., Valenti P., Musci G., Cutone A. (2023). To Boost or to Reset: The Role of Lactoferrin in Energy Metabolism. Int. J. Mol. Sci..

[44] Ianiro G., Rosa L., Bonaccorsi di Patti M.C., Valenti P., Musci G., Cutone A. (2023). Lactoferrin: From the structure to the functional orchestration of iron homeostasis. Biometals.

[45] Bolesławska I., Bolesławska-Król N., Jakubowski K., Przysławski J., Drzymała-Czyż S. (2025). Lactoferrin—A Regulator of Iron Homeostasis and Its Implications in Cancer. Molecules.

[46] Zang L., Morikane D., Shimada Y., Tanaka T., Nishimura N. (2011). A novel protocol for the oral administration of test chemicals to adult zebrafish. Zebrafish.

